# Observing real-time images during ultrasound-guided procedures improves patients’ experience

**DOI:** 10.1093/rheumatology/kev368

**Published:** 2015-10-22

**Authors:** Ilfita Sahbudin, Jason Bell, Kanta Kumar, Karim Raza, Andrew Filer

**Affiliations:** ^1^Department of Rheumatology, Sandwell and West Birmingham Hospitals NHS Trust, Birmingham, UK,; ^2^University Hospitals Birmingham NHS Foundation Trust, Queen Elizabeth Hospital Birmingham, Birmingham, UK,; ^3^Rheumatology Research Group, Institute of Inflammation and Ageing, University of Birmingham, Birmingham, UK and; ^4^Faculty of Medical and Human Sciences, University of Manchester, Manchester, UK

Rheumatology key message

- US-guided injection improves patients’ experience with this intervention, which may contribute to improved response rates.

Sir, US-guided intra-articular and soft tissue steroid injections are common procedures in rheumatology and are taking the place of fluoroscopic and CT-guided injections. Little is known about patients’ views about US-guided steroid injections despite several efficacy studies comparing blind to US-guided injections [1–4]. In the obstetric and gynaecological specialties, patients’ views related to sonography examinations are well documented [5,6]. We conducted a survey to quantitatively capture data relating to patients’ views of US-guided procedures. Ethical approval was not required from the National Health Service (NHS) because this work was considered to be a service evaluation in the NHS Trust in which it took place.

Fifty questionnaires that included balanced Likert scale questions were distributed to rheumatology patients who underwent a US-guided procedure between January 2011 and January 2012. Survey receipt was concluded in April 2012 relating to a post-injection period ranging from 4 to 16 months. Of the 50 questionnaires distributed, 30 (60%) were returned and 26 (50%) were completed and included for data analysis (for the full questionnaire, see Supplementary data, available at *Rheumatology* Online).

A rheumatology consultant with US experience (A.F.) performed all the procedures. During the procedure the sonographer explained to the patient the anatomical features of the diseased target site, power Doppler activity and dynamic needle progression through the superficial tissues into the target site. A total of 34 joints/tendon regions were injected in 26 patients (hand, n = 12; wrist, n = 9; elbow, n = 2; knee, n = 3; ankle, n = 3; feet, n = 5). All procedures were part of the patients’ normal care pathways.

All patients felt that seeing the US images was very helpful or helpful in understanding the procedure (Fig. 1A). Eighty-eight per cent of the patients felt that their levels of worry or anxiety were better or much better as a result of being able to see a US image of the problem area before and during the procedure (Fig. 1C). Ninety-two per cent of patients (24/26) felt that observing the US images in real-time helped with the process of having an injection. Of these 24 patients, 67% felt that observing the US images gave them additional information that helped to improve their understanding of the procedure, 54% of patients felt that the precise area that was causing the pain had been identified and 75% of patients felt that the injection would be aimed at the area causing the pain. If recommended, 95% of patients were very likely or somewhat likely to undergo a further US-guided procedure on the same joint or another inflamed joint (Fig. 1D). Among those who had had a non-guided injection previously (n = 19), 66% of patients felt that US-guided injections were much more effective or somewhat more effective compared with traditional injections (Fig. 1E). Overall, 58% of patients felt that their US-guided injections were much more effective or more effective compared with their expectations (Fig. 1B).

**Fig. 1:**
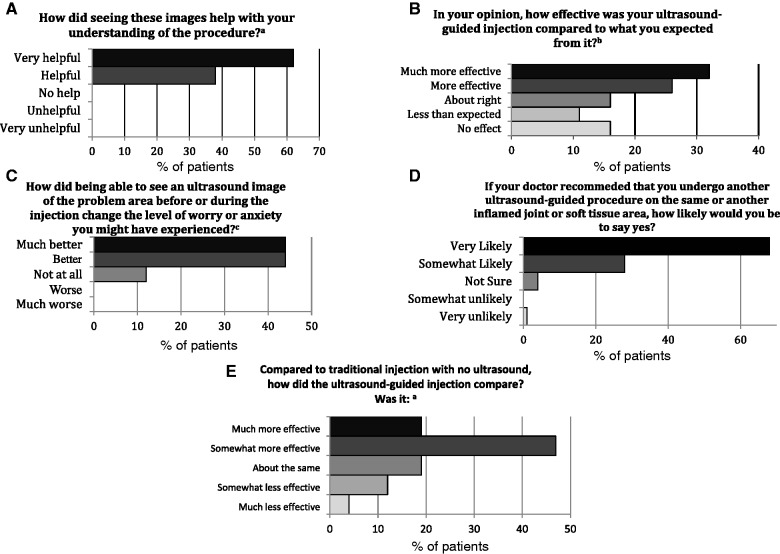
Patients’ views related to observing US images during procedures ^a^n = 26, ^b^n = 19, ^c^n = 25.

This pilot study has some limitations. No validated psychometric questionnaire was available for this specialized purpose, therefore we used a non-validated questionnaire to obtain retrospective views of patients. Furthermore, such a retrospective survey is vulnerable to response bias, potentially enhancing the number of overtly positive or negative responses.

Observing US images during the procedure improved the overall experience of this intervention. Observing the US images in real-time improved patients’ understanding and tolerability of the procedure and reduced patients’ anxiety. This is consistent with a randomised controlled study that suggested US guidance improved pain scores (p < 0.001) and overall response rate (p < 0.01) compared with traditional palpation-guided injection [7]. Anxiety level has been shown to be the strongest negative predictor of poor outcome following facet joint injections [8], indicating that the patient’s level of anxiety affects treatment response.

A larger study is required to confirm our preliminary findings that US-guided injection improves the tolerability of the procedure and reduces patients’ anxiety during the procedure. Further issues that require investigation include whether visualising real-time images during US scanning improves patients’ understanding of disease pathology, which could lead to indirect benefits such as improving therapy adherence and improved pain management strategy.

*Funding*: Arthritis Research UK (Grant 17767) provided funding for the US Unit.

*Disclosure statement*: The authors have declared no conflicts of interest.

## Supplementary data

Supplementary data are available at *Rheumatology* Online.

Supplementary Data
