# Supplementary material for: Observing real-time images during ultrasound-guided procedures improves patients’ experience
Source: Rheumatology (Oxford). 2015 Oct 22;55(3):585–6. doi: 10.1093/rheumatology/kev368 (PMC4746432; doi:10.1093/rheumatology/kev368)
Supplement: Supplementary Data [file supp_55_3_585__index.html]

Observing real-time images during ultrasound-guided procedures improves patients’ experience — Observing real-time images during ultrasound-guided procedures improves patients’ experience — Supplementary Data 

# Observing real-time images during ultrasound-guided procedures improves patients’ experience

## Supplementary Data

files

- Supplementary Data - doc file
