# Supplementary material for: Observing real-time images during ultrasound-guided procedures improves patients’ experience
Source: Rheumatology (Oxford). 2015 Oct 22;55(3):585–6. doi: 10.1093/rheumatology/kev368 (PMC4746432; doi:10.1093/rheumatology/kev368)
Supplement: Supplementary Data [file supp_kev368_rhe-15-0606-File002.doc]

**Ultrasound guided injection: Patient questionnaire**

| **Introduction**  Within the past few months, you will have had an injection to a joint or soft tissue such as a tendon performed using ultrasound guidance.  We are interested in finding out how effective, comfortable and acceptable to patients this approach to giving injection is.  We would like to ask for a minute or two of your time to complete this short questionnaire, placing ticks or crosses in the boxes or writing your thoughts where asked. We would be very grateful if you could then return it in the reply-paid envelope supplied. | | |
| --- | --- | --- |
| No | Question | Answer |
| 1 | Your injection may have occurred some months ago now, so long ago that you cannot remember anything about it. If this is the case, then please tick this box and return the questionnaire. Without completing the remainder of the questions, thank you! | I can’t remember my injection!  If you can remember anything about the injection, then please continue to question 2. If you have had more than one injection using ultrasound, then try to give your overall opinion. |
| 2 | Roughly how long ago did your injection take place? | Less than 2 months  2-4 months  4-6 months  6 months to 1 year  Over 1 year |
| 3 | Please say which joint or soft tissue was injected? | (free text answer) |
| 4 | During the scan you were shown ultrasound images of the problem area before or during the injection.  How did seeing these images help with your understanding of the procedure? | Very helpful  Unhelpful  No help  Helpful  Very helpful |
| 5 | Injections may be painful, and often we become anxious before or during the procedure.  How did being able to see an ultrasound image of the problem area before or during the injection change the level of the anxiety you might have experienced? | Much worse  Worse  Not at all  Better  Much Better |
| 6 | Do you think that being able to see what is happening using ultrasound helps with the process of having an injection? | Yes  No  If yes, in what way? (Tick one or more)  Identifying the precise area which is causing the pain.  Giving extra information so that you understand better what is happening.  Decreasing the level of anxiety about an injection.  Knowing that the injection will be aimed at the area causing the pain  Please write down any other thoughts you have about whether using ultrasound helps with injections (or not). |
| 7 | In your opinion, how effective was your ultrasound guided injection compared to what you expected from it? | No effect  Less than expected  About right  More Effective  Much more effective |
| 8 | In your opinion, how long did the effect of the injection last? | No effect  Less than 1 week  1-4 weeks  1-2 months  Still effective |
| 9 | If your doctor recommended that you undergo another ultrasound guided procedure on the same or another inflamed joint or soft tissue area, how likely would you be to say yes? | Very Unlikely  Somewhat Unlikely  Not Sure  Somewhat Likely  Very Likely |
| 10 | Are there any ways in which you think we could improve the procedure in terms of planning, information given before and during the procedure, or care during and after the procedure? | Please give any specific comments here: |
| 11 | Have you had a “traditional” injection before where the doctor or nurse specialist did not use ultrasound to examine the area and direct the injection ( a”blind” injection)? | Yes  No  If no, then please put this questionnaire in the envelope provided and post it back, thank you! |
| 12 | Compared to ‘traditional’ injection with no ultrasound, how did the ultrasound guided injection compare? Was it: | Much less effective  Somewhat less effective  About the same  Somewhat more effective  Much more effective |
| 13 | In general do you think ultrasound guided injections are better, or worse than traditional injections without ultrasound? If so, please write down why: | Please give any specific comments here: |

Published with permission from Dr Andrew Filer.
